# Risk factors combine in a complex manner in assessment for macrosomia

**DOI:** 10.1186/s12889-023-15195-9

**Published:** 2023-02-07

**Authors:** Yi-Wen Wang, Yan Chen, Yong-Jun Zhang

**Affiliations:** Department of Neonatology, Xinhua Hospital, Shanghai Jiao Tong University School of Medicine, 1665 Kongjiang Road, 200092 Shanghai, China

**Keywords:** Combined effect, Risk factor, Macrosomia

## Abstract

**Background:**

Macrosomia is a serious public health concern. This study aimed to examine the combined effects of various risk factors on macrosomia.

**Methods:**

The China Labor and Delivery Survey was a multicenter cross-sectional study that included 96 hospitals. Logistic regression analysis was performed to examine the combined effects of the risk factors for macrosomia. The population attributable risk percentage (PAR%) was calculated for the risk factors.

**Results:**

A total of 64,735 live births, including 3,739 neonates with macrosomia, were used for the analysis. The weighted prevalence of macrosomia was 5.8%. Pre-pregnancy overweight/obesity, diabetes, and gestational hypertension have a synergistic effect on increasing the rate of macrosomia in mothers aged < 36 years. The highest odds ratio (36.15, 95% CI: 34.38–38.02) was observed in female fetuses whose mothers had both gestational hypertension and diabetes. However, in mothers aged ≥ 36 years, the synergistic effect of gestational hypertension and other factors did not exist, and the risk for macrosomia was reduced by 70% in female fetuses of mothers with both gestational hypertension and overweight/obesity. Pre-pregnancy risk factors (pre-pregnancy overweight/obesity and advanced maternal age) contributed the most to macrosomia (23.36% of the PAR%), and the single largest risk factor was pre-pregnancy overweight/obesity (17.43% of the PAR%).

**Conclusion:**

Macrosomia was related to several common, modifiable risk factors. Some factors have combined effects on macrosomia (e.g., pre-pregnancy overweight/obesity and diabetes), whereas gestational hypertension varies by maternal age. Strategies based on pre-pregnancy risk factors should be given more attention to reduce the burden of macrosomia.

**Supplementary Information:**

The online version contains supplementary material available at 10.1186/s12889-023-15195-9.

## Background

Birth weight and rate of macrosomia have increased over the past four decades in many countries [1–4]. Macrosomia significantly increases the risk of maternal complications, such as postpartum hemorrhage and emergency cesarean section, and is predisposed to a variety of adverse offspring outcomes, including shoulder dystocia and birth asphyxia [5, 6]. In the long term, infants at the highest end of the distribution for weight or body mass index are more likely to be obese in childhood, adolescence, and early adulthood than other infants [7], and they are at greater risk of metabolic complications and cardiovascular disease later in life [8, 9].

Several risk factors have been reported to be related to macrosomia, such as maternal age and height, obesity, high parity, post-term pregnancy, male fetal sex, and diabetes [10]. Among these risk factors, a high pre-pregnancy body mass index (BMI) is an important risk factor for macrosomia [11]. It was reported that pre-pregnancy overweight was associated with a 2.29-fold risk of macrosomia [12], which might be due to increased placental transport of amino acids and other nutrients [13]. Meanwhile, the untreated gestational diabetes group had increased rates of macrosomia compared with normoglycemic controls (28.7% versus 13.7%) [14]. In addition, Xiong et al. found that the rate of large-for-gestational-age neonates was significantly higher in women with gestational hypertension (4.5%) than in those with normal blood pressure (2.2%) [15]. Nevertheless, the situation is complicated by the fact that in the real world, pre-pregnancy overweight/obesity, gestational diabetes, gestational hypertension, and other risk factors often coexist in one individual. For example, it is accepted that high maternal BMI may increase the rate of gestational diabetes, another well-known risk factor for macrosomia, in which case both can synergistically augment the risk of macrosomia [11, 12]. Thus, it would be more valuable to explore how two or more risk factors in combination change the prevalence of macrosomia compared to a single risk factor. To date, few studies on it have been conducted. Therefore, for the well-being of women and their offspring, it is pivotal to identify potentially modifiable risk factors and to investigate and quantify their joint effect on macrosomia, which may help in the development of practical guidelines for the prevention of fetal overgrowth.

## Methods

### Aim

We analyzed data from the China Labor and Delivery Survey to investigate the prevalence and risk factors for macrosomia and further examine the combined effects of the latter on macrosomia.

### Study setting and design

The China Labor and Delivery Survey was a multicenter cross-sectional study conducted throughout the country between March 1, 2015, and December 31, 2016. Hospitals with 1,000 or more deliveries per year were eligible for inclusion. Depending on the annual delivery volume, 5–10 consecutive weeks were randomly chosen over a 12-month period as the study window. Within the chosen weeks, all births delivered at ≥ 24 completed weeks of gestation or with a birthweight of ≥ 500 g were included. Additionally, medical records and extracted information on maternal sociodemographic characteristics, pregnancy and labor complications, pregnancy and medical histories, and perinatal outcomes were retrieved by trained staff. The data management system was programmed with built-in logic checks to validate the consistency of related variables and plausible values. A detailed description of sampling and data management has been published previously [16].

**Fig. 1:**
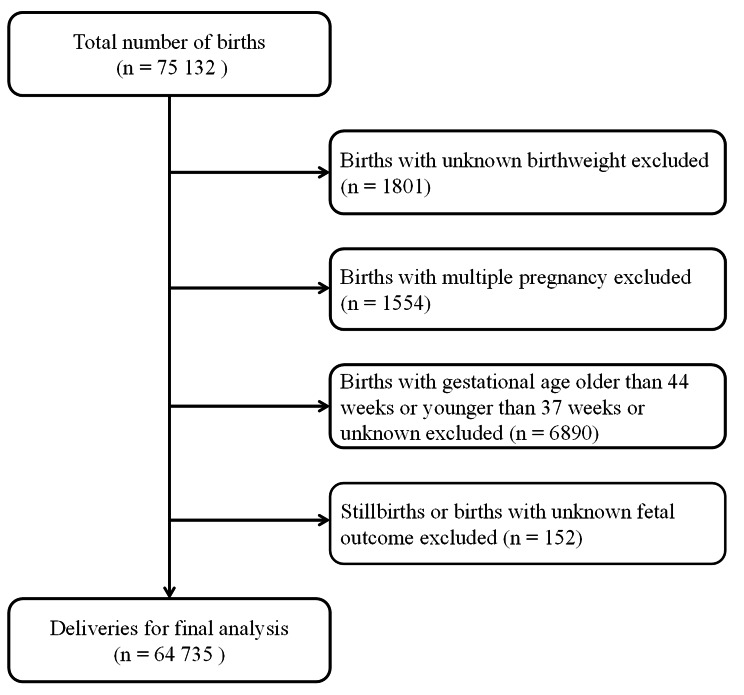
Flow chart

Ninety-six hospitals and 75,132 births distributed in 24 (out of 34) provinces, municipalities, and autonomous regions in China were enrolled. We excluded births with unknown birthweight (n = 1,801), multiple pregnancies (n = 1,554), gestational age (GA) > 44 weeks or < 37 completed weeks or unknown (n = 6,890), and births with unknown fetal outcomes or stillbirths (n = 152), leaving 64,735 live births for the final analysis (Fig. 1). This study was approved by the Ethics Review Board of the Xinhua Hospital Affiliated to the Shanghai Jiao Tong University School of Medicine (XHEC–C–2015–006), the World Health Organization (WHO) Research Ethics Review Committee (HRP Study A65899), and participating hospitals. All methods in this study were performed in accordance with Declaration of Helsinki. Since this was a cross-sectional, observational study and only anonymous clinical information was collected, the consent to participate was deemed unnecessary according to the Ethics Review Board of the Xinhua Hospital Affiliated to the Shanghai Jiao Tong University School of Medicine.

### Definitions

Macrosomia was defined as a birthweight of > 4,000 g [17]. GA was ascertained based on the last menstrual period or ultrasound dating in the first trimester if the date of the last menstrual period was uncertain. Hospital levels are officially determined by local governments [18].

Maternal pre-pregnancy BMI (defined as weight in kilograms divided by the square of height in meters [kg/m^2^]) was categorized as follows: underweight (< 18.5 kg/m^2^), normal (18.5–24.9 kg/m^2^), and overweight or obesity (≥ 25 kg/m^2^), in accordance with the WHO BMI classification [19]. We categorized the maternal education level as low (illiterate, primary school, and junior school), middle (high school, technical school, and junior college), and high (college or higher). We grouped maternal age into ≤ 20 years, 20–35 years, and ≥ 36 years. Post-term pregnancy was defined as a pregnancy lasting longer than 42 gestational weeks. Maternal diseases included pre-pregnancy diabetes mellitus, heart and renal disease, thyroid disease, gestational diabetes mellitus, and hypertensive disorders in pregnancy [including gestational hypertension, preeclampsia, eclampsia, and hemolysis, elevated liver enzymes, and low platelets syndrome (HELLP syndrome)]. Pre-pregnancy diabetes mellitus was diagnosed (a) if the woman was diagnosed with diabetes before pregnancy; or (b) if the fasting plasma glucose ≥ 7 mmol/L or 2-h post load plasma glucose ≥ 11.1 mmol/L during 75 g oral glucose tolerance test or hemoglobin A1c ≥ 6.5%; or (c) with hyperglycemia symptoms or hyperglycemic crisis along with random plasma glucose ≥ 11.1mmol/L [20]. Gestational diabetes mellitus was diagnosed when at least one abnormal plasma glucose value was determined as ≥ 5.1mmol/L (fasting), ≥ 10.0 mmol/L (1 h), and/or ≥ 8.5 mmol/L (2 h) by 75 g oral glucose tolerance test at 24 to 28 weeks of gestation for all women not previously found to have overt diabetes according to International Association of Diabetes and Pregnancy Study Group’s criteria [20, 21]. Women with hypertensive disorders in pregnancy were diagnosed based on the definitions published in 2013 by the American College of Obstetricians and Gynecologists [22]. Gestational hypertension was defined as systolic blood pressure ≥ 140 mmHg or a diastolic blood pressure ≥ 90 mmHg, or both, on two occasions at least 4 h apart after 20 gestational weeks, but without proteinuria, in a woman with a previously normal blood pressure. Preeclampsia was defined as systolic blood pressure ≥ 140 mmHg or diastolic blood pressure ≥ 90 mmHg, measured on at least two occasions after 20 weeks with proteinuria. In the absence of proteinuria, preeclampsia is diagnosed as hypertension in association with thrombocytopenia, impaired liver function, the new development of renal insufficiency, pulmonary edema, or new-onset cerebral or visual disturbances. Eclampsia is diagnosed by new-onset tonic-clonic, focal, or multifocal seizures in a patient with preeclampsia without other causative conditions, such as epilepsy, intracranial hemorrhage, cerebral arterial ischemia, infarction, or drug use.

### Statistical analysis

The 2016 China Statistical Year book provides the number of deliveries in each province [23]. The annual number of births in each province was stratified by the hospital levels. Each birth was assigned a weight based on the inverse probability weighting, taking into account the number of births in the province with the same hospital level and the number of records reviewed in the hospital at the same hospital level [16]. Logistic regression analysis was performed to examine risk factors for macrosomia. We adjusted the analysis of the risk factors for macrosomia according to hospital level, hospital type, maternal age, race, mother’s education level, pre-pregnancy BMI, parity, maternal diseases, infant sex, and post-term pregnancy (i.e., GA ≥ 42 weeks). As potential differences may exist among hospitals and provinces based on preliminary analysis (null models), we conducted generalized linear mixed models with a random effect for hospital-level clustering. Each hospital stood for one unit of analysis (level two); mother-neonate pairs nested within the hospital were the analysis unit at level one. We further examined the risks of delivering macrosomic babies in individuals with different combinations of risk factors with odds ratios (ORs) for macrosomia greater than 1.2, such as maternal age, infant sex, pre-pregnancy BMI, maternal pre-pregnancy diabetes mellitus or gestational diabetes, and gestational hypertension. We grouped our population into 32 subgroups based on age (< 36 and ≥ 36 years), infant sex (male or female), maternal pre-pregnancy diabetes mellitus or gestational diabetes (yes or no), normotensive or gestational hypertension, and pre-pregnancy BMI (< 25 kg/m^2^ or ≥ 25 kg/m^2^). We analyzed the ORs of the subgroups for macrosomia using logistic regression analysis.

The population attributable risk percentage (PAR%) [24] was calculated for risk factor to assess the proportion of macrosomia that could potentially be prevented if risk factors were removed. PAR% was interpreted in this study as the percentage incidence of macrosomia in the population that would be removed if the disease conditions (e.g., diabetes mellitus) were eliminated. We used SPSS version 22.0 (IBM, Somers, NY) and Origin 2021 (Origin Lab Co., Northampton, MA, USA) for statistical analyses and plots.

## Results

### The risk factors for macrosomia in China

**Fig. 2:**
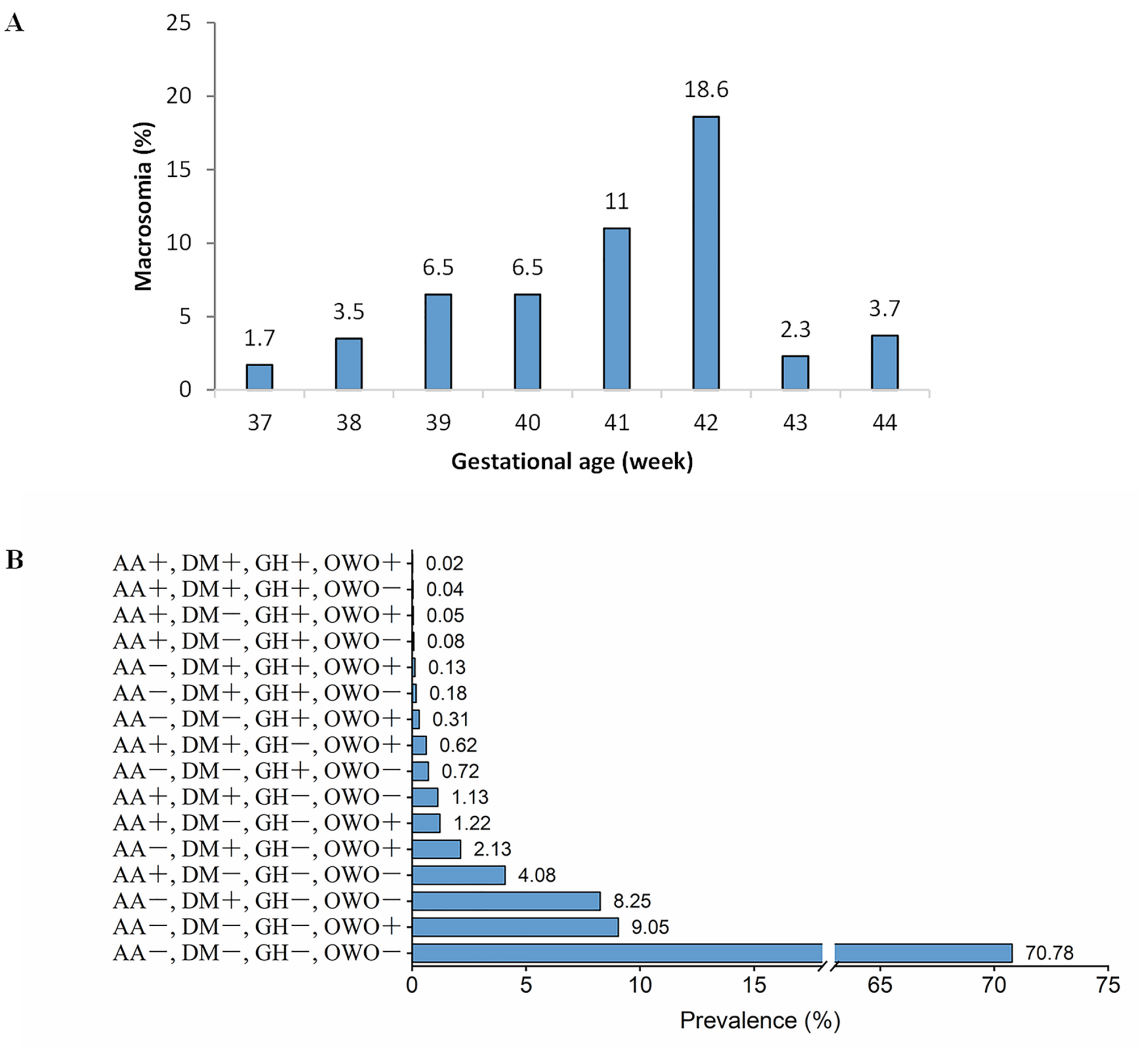
**A** The prevalence of macrosomia according to gestational age. **B** The proportion by clinical characteristics. (AA+: With advanced age (≥ 36 years old), AA-: Without advanced age (< 36 years old), DM+: With pre-pregnancy diabetes mellitus or gestational diabetes, DM-: Without pre-pregnancy diabetes mellitus and gestational diabetes, OWO+: With pre-pregnancy overweight or obesity (body mass index ≥ 25 kg/m^2^), OWO-: Without pre-pregnancy overweight and obesity (body mass index < 25 kg/m^2^), GH+: With gestational hypertension, GH-: Normotensive)

Among the 64,735 subjects, 3,739 had a birth weight of > 4,000 g. The weighted prevalence of macrosomia was 5.8%. The weighted prevalence of macrosomia was 1.7%, 3.5%, 6.5%, 6.5%, 11%, 18.6%, 2.3%, and 3.7% in neonates with GA of 37, 38, 39, 40, 41, 42, 43, and 44 weeks, respectively ( Fig. 2A). Several factors were significantly associated with an increased risk for macrosomia (Table 1). Among these risk factors, advanced age (≥ 36) [adjusted OR (aOR) 1.85; 95% confidence interval (CI) 1.83–1.87], pre-pregnancy overweight/obesity (≥ 25 kg/m^2^) (aOR 2.15; 95% CI 2.14–2.17), pre-pregnancy diabetes mellitus (aOR 2.73; 95% CI 2.66–2.79), gestational hypertension (aOR 2.10; 95% CI 2.05–2.14), gestational diabetes (aOR 1.44; 95% CI 1.42–1.45), and infant male sex (aOR 1.56; 95% CI 1.55–1.57) were important risk factors for macrosomia.

**Table 1:** Distribution of characteristics and their associations with macrosomia: China, 2015–2016

Characteristics	Weighted number of live births (%) n = 7,877,832	Fetal macrosomia		
		**Weighted number of fetal macrosomia** **(per 100 live births)**	OR (95%CI)*	**aOR (95%CI)** * †
**Hospital level**				
Secondary hospital	4,552,808 (57.8)	262,177 (5.8)	1 (Ref)	1 (Ref)
Tertiary hospital	3,325,024 (42.2)	195,973 (5.9)	1.03 (1.02, 1.03)	1.01 (0.99, 1.02)
**Hospital type**				
General hospital	4,372,305 (55.5)	245,512(5.6)	1 (Ref)	1 (Ref)
Maternity hospital	3,505,527 (44.5)	212,638 (6.1)	1.09 (1.08, 1.09)	0.93 (0.92, 0.94)
**Maternal age**				
≤ 19	178,325 (2.3)	2333 (1.3)	0.23 (0.22, 0.24)	0.18 (0.17, 0.19)
20–35	7,093,762 (90.0)	389,227 (5.5)	1 (Ref)	1 (Ref)
≥ 36	605,745 (7.7)	66,590 (11.0)	2.13 (2.11, 2.15)	1.85 (1.83, 1.87)
**Race**				
Han	7,618,711 (96.7)	436,596 (5.7)	1 (Ref)	1 (Ref)
Other	259,122 (3.3)	21,554 (8.3)	1.49 (1.47, 1.51)	2.15 (2.12, 2.18)
**Mother’s education level** ^**a**^				
Low	2,664,510 (37.5)	161,231 (6.1)	0.98 (0.97, 0.99)	1.18 (1.17, 1.19)
Middle	2,780,083 (39.2)	171,643 (6.2)	1 (Ref)	1 (Ref)
High	1,655,962 (23.3)	87,031 (5.3)	0.84 (0.84, 0.85)	0.89 (0.88, 0.90)
**Pre-pregnancy BMI**				
Underweight (< 18.5 kg/m^2^)	748,712 (13.9)	13,542 (1.8)	0.29 (0.29, 0.30)	0.27 (0.27, 0.28)
Normal (18.5–24.9 kg/m^2^)	3,873,918 (72.1)	229,420 (5.9)	1 (Ref)	1 (Ref)
Overweight or obesity (≥ 25 kg/m^2^)	746,698 (13.9)	96,861 (13.0)	2.37 (2.35, 2.39)	2.15 (2.14, 2.17)
**Parity**				
0	2,626,867 (33.4)	130,452 (5.0)	1 (Ref)	1 (Ref)
≥ 1	5,228,683 (66.6)	327,079 (6.3)	1.28 (1.27, 1.29)	1.16 (1.15, 1.17)
**Pre-pregnancy diabetes mellitus**				
No	7,749,556 (99.2)	442,233 (5.7)	1 (Ref)	1 (Ref)
Yes	65,331 (0.8)	13,840 (21.2)	4.44 (4.36, 4.53)	2.73 (2.66, 2.79)
**Heart disease**				
No	7,835,668 (99.7)	456,991 (5.8)	1 (Ref)	1 (Ref)
Yes	24,944 (0.3)	782 (3.1)	0.52 (0.49, 0.56)	0.15 (0.13, 0.18)
**Renal disease**				
No	7,854,448 (99.9)	456,819 (5.8)	1 (Ref)	1 (Ref)
Yes	9192 (0.1)	268 (2.9)	0.49 (0.43, 0.55)	0.32 (0.28, 0.37)
**Thyroid disease**				
No	7,452,065 (97.0)	432,716 (5.8)	1 (Ref)	1 (Ref)
Yes	226,614 (3.0)	9334 (4.1)	0.70 (0.68, 0.71)	0.65 (0.64, 0.67)
**Hypertensive disorders in pregnancy**				
No	7,514,731 (97.1)	428,996 (5.7)	1 (Ref)	1 (Ref)
Gestational hypertension	119,050 (1.5)	13,561 (11.4)	2.12 (2.09, 2.16)	2.10 (2.05, 2.14)
Preeclampsia /HELLP/ eclampsia	108,030 (1.4)	7344 (6.8)	1.21 (1.18, 1.23)	0.87 (0.84, 0.90)
**Gestational diabetes**				
No	6,808,705 (89.6)	369,983(5.4)	1 (Ref)	1 (Ref)
Yes	792,093 (10.4)	82,810 (10.5)	2.03 (2.02, 2.05)	1.44 (1.42, 1.45)
**Sex**				
Female	3,691,641 (46.9)	159,503 (4.3)	1 (Ref)	1 (Ref)
Male	4,182,912 (53.1)	298,047 (7.1)	1.70 (1.69, 1.71)	1.56 (1.55, 1.57)
**Post-term pregnancy**				
No	7,838,836 (99.5)	452,525 (5.8)	1 (Ref)	1 (Ref)
Yes	38,997 (0.5)	5625 (14.4)	2.75 (2.67, 2.83)	3.75 (3.62, 3.90)

^a^ Mother’s education level: low (illiterate, primary school, and junior school), middle (high school, technical school, and junior college), and high (college or higher degree)

BMI, body mass index

HELLP: hemolysis, elevated liver enzymes, and low platelets syndrome

*Adjusted for sampling distribution

†adjusted for all other covariates in the model

In generalized linear mixed models with a random effect for the hospital-level clustering (Supplementary Table 1), the modifiable risk factors and their odds ratios for macrosomia were similar to those identified by the multivariable logistic regression models. Finally, important risk factors including maternal age, infant sex, pre-pregnancy BMI, maternal pre-pregnancy diabetes mellitus or gestational diabetes, and gestational hypertension were used in the further analysis.

### The combined effect of risk factors on macrosomia

We further analyzed the combined effects of important risk factors on macrosomia. There were 47,120 subjects with information on risk factors of maternal age, infant sex, pre-pregnancy BMI, maternal pre-pregnancy diabetes mellitus or gestational diabetes, and gestational hypertension. We grouped the participants into different groups based on different combinations of risk factors. The normal population was the group of normotensive mothers aged < 36 years without diabetes and pre-pregnancy overweight /obesity, which accounted for 70.78%. In mothers aged < 36 years with risk factors, the highest proportions were found for mothers with only pre-pregnancy overweight/obesity (9.05%) and diabetes (8.25%), followed by both factors (2.13%). The proportion of mothers with only the risk factor of advanced age (≥ 36 years) was 4.08% (Fig. 2B).

**Fig. 3:**
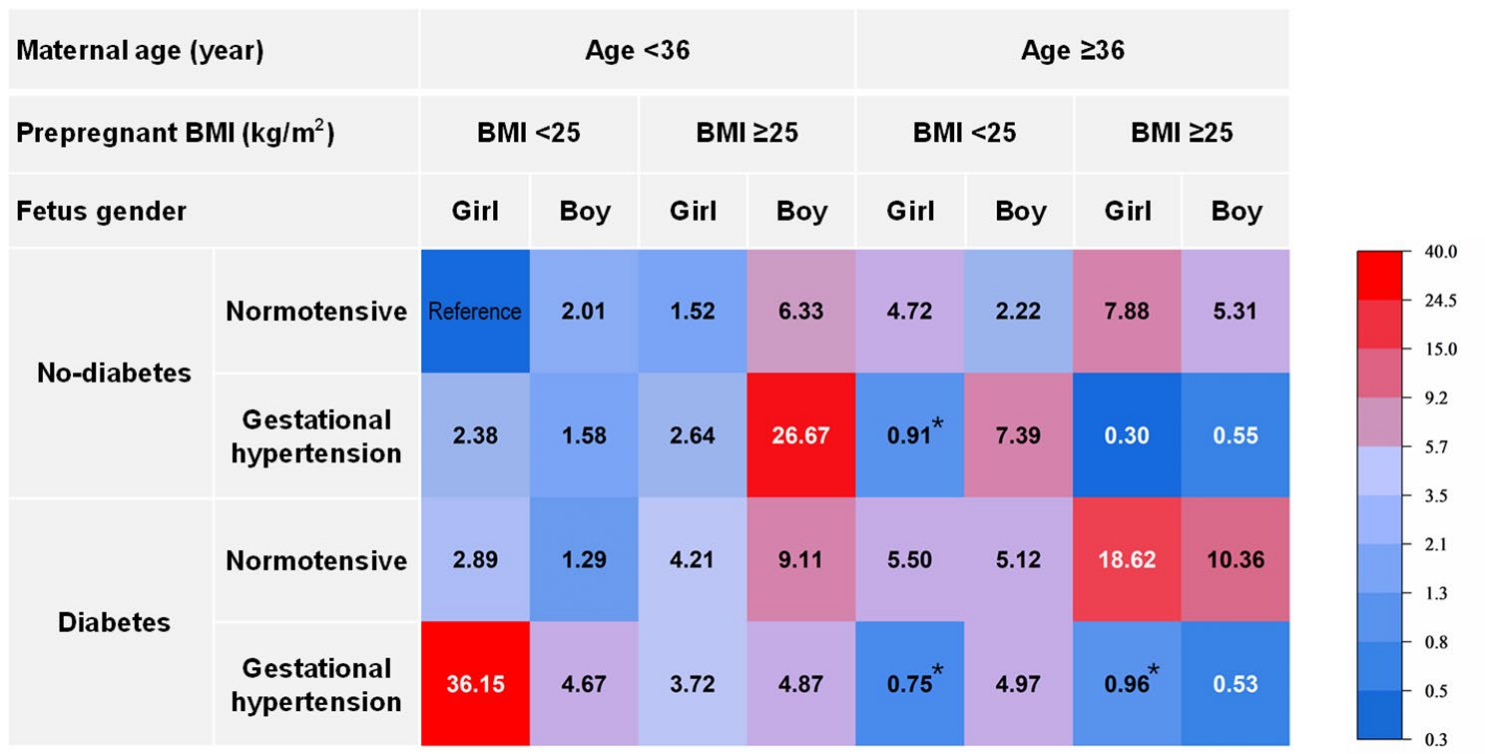
The risks of delivering macrosomic babies in individuals with different combinations of risk factors

The hierarchical analysis showed the different combined effects of risk factors on macrosomia in the groups of mothers aged < 36 years and ≥ 36 years (Supplementary Table 2). Figure 3 presents a heat map illustrating the risks of delivering macrosomia babies in individuals with different combinations of risk factors, including maternal age, infant sex, pre-pregnancy BMI, maternal pre-pregnancy diabetes mellitus or gestational diabetes, and gestational hypertension. Red denotes high risk, and blue denotes low risk. The combined effect of the risk factors for macrosomia was different in the groups of mothers aged < 36 years and ≥ 36 years. In the group of mothers aged < 36 years, all factors had a synergistic effect in increasing the incidence of macrosomia. Among them, the highest OR (36.15; 95% CI 34.38–38.02) was observed in the female fetuses whose mothers had both gestational hypertension and diabetes but without overweight/obesity, followed by the male fetuses whose mothers had both gestational hypertension and overweight/obesity but without diabetes (OR 26.67; 95% CI 25.63–27.76). In the group of mothers aged ≥ 36 years, not all risk factors had a synergistic effect on the risk of macrosomia. The three lowest ORs were found in mothers with gestational hypertension and overweight/obesity. For example, the OR for macrosomia was only 0.30 (95% CI 0.21–0.44) in the female fetuses of mothers with both gestational hypertension and overweight/obesity but without diabetes. Moreover, when all risk factors occurred, the risk for macrosomia fell to 0.53 (95% CI 0.36–0.78).

### The PAR% for risk factors

**Fig. 4:**
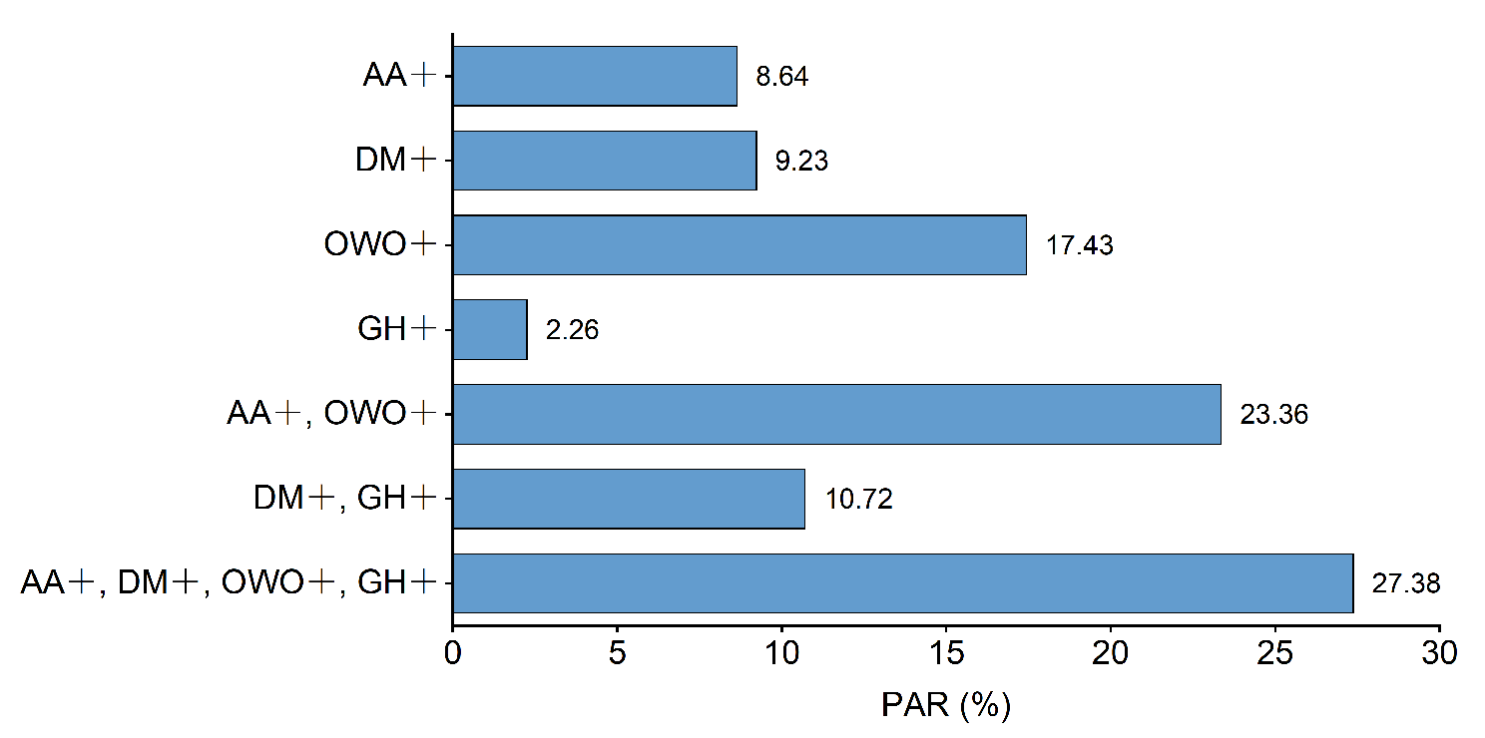
Population attributable risk percentage for macrosomia. (AA+: With advanced age (≥ 36 years old), DM+: With pre-pregnancy diabetes mellitus or gestational diabetes, OWO+: With pre-pregnancy overweight or obesity (body mass index ≥ 25 kg/m^2^), GH+: With gestational hypertension)

The PAR% for modifiable risk factors is shown in Fig. 4. Reducing the incidence of gestational hypertension would lead to a reduction in the incidence of macrosomia by 2.26%, while reducing the incidence of advanced maternal age would lead to a reduction in the incidence of macrosomia by 8.64%. Reducing the incidence of pre-pregnancy diabetes mellitus or gestational diabetes would lead to a reduction in the incidence of macrosomia by 9.23%, and reducing the incidence of pre-pregnancy overweight or obesity would lead to a reduction in the incidence of macrosomia by 17.43%. For pre-pregnancy risk factors, the PAR% related to mothers with advanced maternal age or pre-pregnancy overweight/obesity was as high as 23.36%, while for pregnancy complications, the PAR% related to mothers with diabetes or gestational hypertension was relatively lower, at 10.72%. In the entire study, the PAR% associated with all four risk factors was 27.38%.

## Discussion

We confirmed that macrosomia was related to several common and modifiable risk factors, such as maternal age, pre-pregnancy overweight/obesity, diabetes, and gestational hypertension, which have different effects on macrosomia by combination. For example, pre-pregnancy overweight/obesity combined with diabetes had a much higher risk than their individual risks, while the effects of gestational hypertension varied by maternal age.

In 2007–2008, the prevalence of macrosomia in China was 6.9% [25]. It has decreased to 5.8% in our study, probably due to the changes in sanitation, healthcare and dietary structure, but is still higher than the average of the 23 low-income and middle-income countries surveyed (4.5%) [26].

The synergistic effects of pre-pregnancy overweight/obesity and diabetes on macrosomia are biologically plausible. Yang et al. attributed this to the fact that the former is a strong risk factor for the latter [12]. However, few studies have explored the combined effects of gestational hypertension and other risk factors for macrosomia, and some findings are inconsistent. Xiong et al. reported that gestational hypertension was positively associated not only with large-for-gestational-age infants but also with small-for-gestational-age infants [15], presumably because they did not stratify mothers into different age groups in their study. In the present study, we confirmed that the composite effect of gestational hypertension and other risk factors on macrosomia varied with maternal age. In the group of mothers aged < 36 years, we revealed that gestational hypertension combined with either one or both of diabetes or pre-pregnancy overweight/obesity was associated with a 2.87–14.06-fold increased risk of macrosomia compared with women not having any of overweight/obesity, diabetes, or gestational hypertension (data not shown). In mothers older than 36 years, the synergistic effect of gestational hypertension and other risk factors did not persist. We inferred that the discrepancy in the effect of gestational hypertension on offspring between the two age groups might be related to the onset time of gestational hypertension as well as its severity. It was reported that mothers of advanced age complicated by hypertensive disorders had lower neonatal birthweights and a higher rate of composite adverse neonatal outcomes [27]. Moreover, mothers of advanced age were more likely to have early-onset hypertensive disorders during pregnancy, whereas younger women tended to experience late-onset hypertensive disorders [28]. Hence, in young women, hypertension disorders tend to present later in pregnancy, wherein uteroplacental blood flow may be greater throughout most of their pregnancies until a certain severity of disorder is reached. In this case, the decreased uteroplacental perfusion might be too short in duration to reverse the earlier growth-enhancing effects of high blood flow caused by high blood pressure. In women with advanced age and early-onset hypertension disorders, the early-onset decreased uteroplacental perfusion hampers the delivery of macrosomia.

In the present study, we identified four important modifiable risk factors in the primary prevention of macrosomia: diabetes, advanced maternal age, gestational hypertension, and pre-pregnancy overweight/obesity, with recently reported prevalence of 14.8%, 10.9%, 1.2%, and 12.1%/2.8% reported in China, respectively [29–32]. Strategies such as changing lifestyle patterns and controlling for underlying conditions may control these risk factors, thereby reducing the incidence of macrosomia. For example, diabetes is a risk factor for macrosomia, with a PAR% of 9.23%. For interventions for gestational diabetes, the pooled result of a meta-analysis of four randomized controlled trials [33] suggests that diet counseling or insulin treatment (if needed) for women with the disorder is associated with a lower risk of macrosomia than usual care (OR 0.38; 95% CI 0.30–0.49). As for advanced maternal age, the PAR% was 8.64%. In China, the fertility age showed a significant growth trend. The average age of first birth has increased from 23.49 to 1995 to 25.78 years old in 2012, and the average age of second birth has risen from 26.73 to 29.61 years old [34]. After the implementation of the universal two child policy, a Chinese national surveillance data showed that births to women with advanced age increased from 7.8% to 2012 to 10.9% in 2016 [30]. Thereby, the impact of advanced maternal age on offspring outcomes deserves concerning. With respect to gestational hypertension, the PAR% was 2.26%. Several factors are accepted to be associated with the development of gestational hypertension. Besides factors such as endothelial dysfunction of maternal vessels, pre-pregnancy BMI was also associated with gestational hypertension [35, 36]. In this case, we speculated that good management of pre-pregnancy BMI to reduce gestational hypertension may also help to decline the incidence of macrosomia. Our findings on the association between pre-pregnancy overweight/obesity and macrosomia also seemed to lend support the above. Pre-pregnancy overweight/obesity was a stronger contributor to macrosomia, with a PAR% of 17.43%. Thus, preventing pre-pregnancy overweight or obesity during preparation for pregnancy, such as changing lifestyle patterns, deserves more attention to decrease the incidence of macrosomia. More importantly, the PAR% related to mothers with pre-pregnancy overweight/obesity or advanced maternal age was as high as 23.36%, much higher than the PAR% related to mothers with diabetes or gestational hypertension (10.72%), indicating that controlling pre-pregnancy risk factors, especially pre-pregnancy overweight/obesity, is more effective in reducing the incidence of macrosomia than controlling diseases during pregnancy, which might be helpful in implementing strategies to prevent macrosomia.

### Limitations

Our study has several limitations. First, because the study was not designed to screen all women for diabetes, underdiagnosis could have occurred in some settings, and some cases of diabetes might have been missed. However, the risk of diabetes on macrosomia was similar to previously reported estimates [26]. Second, we did not have information on gestational weight gain, which is associated with macrosomia [5], thus, its confounding and independent effects are unclear. Third, we lacked information regarding the time of occurrence of gestational hypertension. Fourth, in this large national cross-sectional study, there were indeed some variables with missing data, including maternal pre-pregnancy BMI, mother’s education level, parity, infant sex, and maternal diseases, with the most missing variable being maternal pre-pregnancy BMI (24.79%). Therefore, we performed an analysis using data from East China, where the missing information above could be reduced to 10.55%. That is, about 90% of subjects with complete data on variables were used for analysis. The risk factors for macrosomia were approximate to the national cross-sectional study (Supplementary Table 3).

## Conclusion

To the best of our knowledge, our study is the first to assess the combined effects of multiple risk factors for macrosomia. The combined effects of gestational hypertension and other risk factors for macrosomia vary with maternal age. In mothers aged < 36 years, gestational hypertension, diabetes, and pre-pregnancy overweight/obesity had a synergistic effect on the risk of macrosomia. However, in the groups of mothers aged ≥ 36 years, synergistic effects of gestational hypertension and other factors on the risk for macrosomia did not exist. Furthermore, pre-pregnancy overweight/obesity is an important contributor to macrosomia, especially in combination with advanced maternal age. Thus, more attention should be paid to strategies based on pre-pregnancy risk factors to reduce the burden of macrosomia.

## Electronic supplementary material

Below is the link to the electronic supplementary material.

Supplementary Material 1

## Data Availability

The datasets generated during and/or analyzed during the current study are available from the corresponding author on reasonable request.

## Abbreviations

BMI: Body mass index
WHO: World Health Organization
GA: Gestational age
HELLP: Hemolysis, elevated liver enzymes, and low platelets
OR: Odds ratio
PAR: Population attributable risk
