# Supplementary material for: Risk factors combine in a complex manner in assessment for macrosomia
Source: BMC Public Health. 2023 Feb 7;23:271. doi: 10.1186/s12889-023-15195-9 (PMC9906846; doi:10.1186/s12889-023-15195-9)
Supplement: Supplementary file 1 — Supplementary Material 1 [file 12889_2023_15195_MOESM1_ESM.doc]

Supplementary Table 1 The associations of risk factors with macrosomia in generalized linear mixed model with a random effect for the hospital-level clustering, China, 2015-2016

| **Characteristics** | **Fetal macrosomia** |
| --- | --- |
|  | **aOR (95%CI)** * † |
| **Hospital level** |  |
| Secondary hospital | 1 (Ref) |
| Tertiary hospital | 1.00 (0.59, 1.71) |
| **Hospital type** |  |
| General hospital | 1 (Ref) |
| Maternity hospital | 0.87 (0.55, 1.39) |
| **Maternal age** |  |
| ≤ 19 | 0.21 (0.08, 0.57) |
| 20-35 | 1 (Ref) |
| ≥ 36 | 1.83 (1.26, 2.64) |
| **Race** |  |
| Han | 1 (Ref) |
| Other | 2.50 (1.03, 6.06) |
| **Mother’s education level^a^** |  |
| Low | 1.21 (0.83, 1.77) |
| Middle | 1 (Ref) |
| High | 0.83 (0.71, 0.98) |
| **Pre-pregnancy BMI** |  |
| Underweight (< 18.5 kg/m^2^) | 0.29 (0.15, 0.54) |
| Normal (18.5–24.9 kg/m^2^) | 1 (Ref) |
| Overweight or obesity (≥ 25 kg/m^2^) | 2.12 (1.51, 2.96) |
| **Parity** |  |
| 0 | 1 (Ref) |
| ≥ 1 | 1.20 (0.87, 1.65) |
| **Pre-pregnancy diabetes mellitus** |  |
| No | 1 (Ref) |
| Yes | 2.76 (1.03, 7.39) |
| **Heart disease** |  |
| No | 1 (Ref) |
| Yes | 0.16 (0.04, 0.69) |
| **Renal disease** |  |
| No | 1 (Ref) |
| Yes | 0.28 (0.02, 4.76) |
| **Thyroid disease** |  |
| No | 1 (Ref) |
| Yes | 0.63 (0.33, 1.22) |
| **Hypertensive disorders in pregnancy** |  |
| No | 1 (Ref) |
| Gestational hypertension | 2.14 (1.21, 4.27) |
| Preeclampsia /HELLP/ eclampsia | 0.80 (0.27, 2.38) |
| **Gestational diabetes** |  |
| No | 1 (Ref) |
| Yes | 1.43 (1.14, 1.78) |
| **Sex** |  |
| Female | 1 (Ref) |
| Male | 1.58 (1.01, 2.95) |
| **Post-term pregnancy** |  |
| No | 1 (Ref) |
| Yes | 3.82 (1.02, 15.30) |

^a^ Mother’s education level: low (illiterate, primary school, and junior school), middle (high school, technical school, and junior college), and high (college or higher degree)

BMI, body mass index

HELLP：hemolysis, elevated liver enzymes, and low platelets syndrome

*Adjusted for sampling distribution

†adjusted for all other covariates in the model

Supplementary Table 2 Different combinations of risk factors for macrosomia

| Group | N (%) | OR (95%CI) * |
| --- | --- | --- |
| Maternal age < 36 years, no-diabetes, normotensive, pre-pregnancy BMI < 25 kg/m^2^, female fetus gender | 1648141 (32.56) | 1 (Ref) |
| Maternal age < 36 years, no-diabetes, normotensive， pre-pregnancy BMI < 25 kg/m^2^, male fetus gender | 1934769 (38.22) | 2.01 (1.98, 2.03) |
| Maternal age < 36 years, no-diabetes, normotensive, pre-pregnancy BMI ≥ 25kg/m^2^, female fetus gender | 227898 (4.50) | 1.52 (1.49, 1.55) |
| Maternal age < 36 years, no-diabetes, normotensive, pre-pregnancy BMI ≥ 25kg/m^2^, male fetus gender | 230306 (4.55) | 6.33 (6.24, 6.42) |
| Maternal age < 36 years, no-diabetes, gestational hypertension, pre-pregnancy BMI < 25 kg/m^2^, female fetus gender | 14696 (0.29) | 2.38 (2.23, 2.54) |
| Maternal age < 36 years, no-diabetes, gestational hypertension, pre-pregnancy BMI < 25 kg/m^2^, male fetus gender | 21974 (0.43) | 1.58 (1.48, 1.68) |
| Maternal age < 36 years, no-diabetes, gestational hypertension, pre-pregnancy BMI ≥ 25kg/m^2^, female fetus gender | 5378 (0.11) | 2.64 (2.39, 2.92) |
| Maternal age < 36 years, no-diabetes, gestational hypertension, pre-pregnancy BMI ≥ 25kg/m^2^, male fetus gender | 10198 (0.20) | 26.67 (25.63, 27.76) |
| Maternal age < 36 years, diabetes, normotensive, pre-pregnancy BMI < 25 kg/m^2^, female fetus gender | 218702 (4.32) | 2.89 (2.84, 2.95) |
| Maternal age < 36 years, diabetes, normotensive, pre-pregnancy BMI < 25 kg/m^2^, male fetus gender | 198646 (3.92) | 1.29 (1.26, 1.32) |
| Maternal age < 36 years, diabetes, normotensive, pre-pregnancy BMI ≥ 25kg/m^2^, female fetus gender | 54807 (1.08) | 4.21 (4.09, 4.32) |
| Maternal age < 36 years, diabetes, normotensive, pre-pregnancy BMI ≥ 25kg/m^2^, male fetus gender | 52788 (1.04) | 9.11 (8.91, 9.31) |
| Maternal age < 36 years, diabetes, gestational hypertension, pre-pregnancy BMI < 25 kg/m^2^, female fetus gender | 6294 (0.12) | 36.15 (34.38, 38.02) |
| Maternal age < 36 years, diabetes, gestational hypertension, pre-pregnancy BMI < 25 kg/m^2^, male fetus gender | 2888 (0.06) | 4.67 (4.19, 5.21) |
| Maternal age < 36 years, diabetes, gestational hypertension, pre-pregnancy BMI ≥ 25kg/m^2^, female fetus gender | 2541 (0.05) | 3.72 (3.28, 4.22) |
| Maternal age < 36 years, diabetes, gestational hypertension, pre-pregnancy BMI ≥ 25kg/m^2^, male fetus gender | 3821 (0.08) | 4.87 (4.43, 5.34) |
| Maternal age ≥ 36 years, no-diabetes, normotensive, pre-pregnancy BMI < 25 kg/m^2^, female fetus gender | 101000 (2.00) | 4.72 (4.63, 4.82) |
| Maternal age ≥36 years, no-diabetes, normotensive, pre-pregnancy BMI < 25 kg/m^2^, male fetus gender | 105559 (2.09) | 2.22 (2.16, 2.28) |
| Maternal age ≥ 36 years, no-diabetes, normotensive, pre-pregnancy BMI ≥ 25kg/m^2^, female fetus gender | 30523 (0.60) | 7.88 (7.66, 8.12) |
| Maternal age ≥ 36 years, no-diabetes, normotensive, pre-pregnancy BMI ≥ 25kg/m^2^, male fetus gender | 31241 (0.62) | 5.31 (5.14, 5.49) |
| Maternal age ≥ 36 years, no-diabetes, gestational hypertension, pre-pregnancy BMI < 25 kg/m^2^, female fetus gender | 1923 (0.04) | 0.91 (0.70, 1.19) |
| Maternal age ≥ 36 years, no-diabetes, gestational hypertension, pre-pregnancy BMI < 25 kg/m^2^, male fetus gender | 2160 (0.04) | 7.39 (6.63, 8.22) |
| Maternal age ≥ 36 years, no-diabetes, gestational hypertension, pre-pregnancy BMI ≥ 25kg/m^2^, female fetus gender | 1084 (0.02) | 0.30 (0.21, 0.44) |
| Maternal age ≥ 36 years, no-diabetes, gestational hypertension, pre-pregnancy BMI ≥ 25kg/m^2^, male fetus gender | 1609 (0.03) | 0.55 (0.43, 0.69) |
| Maternal age ≥ 36 years, diabetes, normotensive, pre-pregnancy BMI < 25 kg/m^2^, female fetus gender | 27307 (0.54) | 5.50 (5.31, 5.69) |
| Maternal age ≥ 36 years, diabetes, normotensive, pre-pregnancy BMI < 25 kg/m^2^, male fetus gender | 29942 (0.59) | 5.12 (4.95, 5.30) |
| Maternal age ≥ 36 years, diabetes, normotensive, pre-pregnancy BMI ≥ 25kg/m^2^, female fetus gender | 10701(0.21) | 18.62 (17.89, 19.38) |
| Maternal age ≥ 36 years, diabetes, normotensive, pre-pregnancy BMI ≥ 25kg/m^2^, male fetus gender | 20462 (0.40) | 10.36 (10.03, 10.71) |
| Maternal age ≥ 36 years, diabetes, gestational hypertension, pre-pregnancy BMI < 25 kg/m^2^, female fetus gender | 1325 (0.03) | 0.75 (0.52, 1.07) |
| Maternal age ≥ 36 years, diabetes, gestational hypertension, pre-pregnancy BMI < 25 kg/m^2^, male fetus gender | 474 (0.01) | 4.97 (3.82, 6.45) |
| Maternal age ≥ 36 years, diabetes, gestational hypertension, pre-pregnancy BMI ≥ 25kg/m^2^, female fetus gender | 416 (0.01) | 0.96 (0.67, 1.38) |
| Maternal age ≥ 36 years, diabetes, gestational hypertension, pre-pregnancy BMI ≥ 25kg/m^2^, male fetus gender | 609 (0.01) | 0.53 (0.36, 0.78) |

The mothers with preeclampsia /HELLP(hemolysis, elevated liver enzymes, and low platelets syndrome)/ eclampsia were not inclued in the analysis.

BMI, body mass index

*Adjusted for sampling distribution

Supplementary Table 3 The associations of risk factors with macrosomia in East China, 2015-2016

| **Characteristics** | **Fetal macrosomia** | |
| --- | --- | --- |
|  | **OR (95%CI)*** | **aOR (95%CI)** * † |
| **Hospital level** |  |  |
| Secondary hospital | 1 (Ref) | 1 (Ref) |
| Tertiary hospital | 0.91 (0.90, 0.93) | 1.14 (1.12, 1.16) |
| **Hospital type** |  |  |
| General hospital | 1 (Ref) | 1 (Ref) |
| Maternity hospital | 0.96 (0.95, 0.98) | 0.69 (0.68, 0.70) |
| **Maternal age** |  |  |
| ≤ 19 | 0.18 (0.16, 0.20) | 0.28 (0.25, 0.31) |
| 20-35 | 1 (Ref) | 1 (Ref) |
| ≥ 36 | 1.90 (1.86, 1.94) | 1.80 (1.76, 1.84) |
| **Race** |  |  |
| Han | 1 (Ref) | 1 (Ref) |
| Other | 0.56 (0.52, 0.61) | 0.34 (0.30, 0.38) |
| **Mother’s education level^a^** |  |  |
| Low | 0.76 (0.75, 0.78) | 0.68 (0.67, 0.69) |
| Middle | 1 (Ref) | 1 (Ref) |
| High | 0.65 (0.64, 0.66) | 0.75 (0.73, 0.76) |
| **Pre-pregnancy BMI** |  |  |
| Underweight (< 18.5 kg/m^2^) | 0.11 (0.10, 0.11) | 0.12 (0.11, 0.12) |
| Normal (18.5–24.9 kg/m^2^) | 1 (Ref) | 1 (Ref) |
| Overweight or obesity (≥ 25 kg/m^2^) | 3.22 (3.17, 3.26) | 2.76 (2.72, 2.80) |
| **Parity** |  |  |
| 0 | 1 (Ref) | 1 (Ref) |
| ≥ 1 | 0.93 (0.92, 0.95) | 1.02 (0.98, 1.05) |
| **Pre-pregnancy diabetes mellitus** |  |  |
| No | 1 (Ref) | 1 (Ref) |
| Yes | 9.62 (9.28, 9.96) | 3.58 (3.42, 3.74) |
| **Heart disease** |  |  |
| No | 1 (Ref) | 1 (Ref) |
| Yes | 0.85 (0.75, 0.97) | 0.58 (0.50, 0.68) |
| **Renal disease** |  |  |
| No | NA | NA |
| Yes | NA | NA |
| **Thyroid disease** |  |  |
| No | 1 (Ref) | 1 (Ref) |
| Yes | 0.56 (0.54, 0.58) | 0.61 (0.58, 0.64) |
| **Hypertensive disorders in pregnancy** |  |  |
| No | 1 (Ref) | 1 (Ref) |
| Gestational hypertension | 3.73 (3.62, 3.85) | 3.06 (2.95, 3.17) |
| Preeclampsia /HELLP/ eclampsia | 1.67 (1.57, 1.78) | 0.70 (0.64, 0.75) |
| **Gestational diabetes** |  |  |
| No | 1 (Ref) | 1 (Ref) |
| Yes | 3.19 (3.14, 3.25) | 2.27 (2.22, 2.32) |
| **Sex** |  |  |
| Female | 1 (Ref) | 1 (Ref) |
| Male | 1.92 (1.89, 1.94) | 1.65 (1.63, 1.67) |
| **Post-term pregnancy** |  |  |
| No | 1 (Ref) | 1 (Ref) |
| Yes | 1.43 (1.29, 1.58) | 1.97 (1.75, 2.21) |

^a^ Mother’s education level: low (illiterate, primary school, and junior school), middle (high school, technical school, and junior college), and high (college or higher degree)

BMI, body mass index

HELLP：hemolysis, elevated liver enzymes, and low platelets syndrome

*Adjusted for sampling distribution

†adjusted for all other covariates in the model
